# Intragenomic Profiling Using Multicopy Genes: The rDNA Internal Transcribed Spacer Sequences of the Freshwater Sponge *Ephydatia fluviatilis*

**DOI:** 10.1371/journal.pone.0066601

**Published:** 2013-06-18

**Authors:** Liisi Karlep, Tõnu Reintamm, Merike Kelve

**Affiliations:** Department of Gene Technology, Tallinn University of Technology, Tallinn, Estonia; Texas A&M University, United States of America

## Abstract

Multicopy genes, like ribosomal RNA genes (rDNA), are widely used to describe and distinguish individuals. Despite concerted evolution that homogenizes a large number of rDNA gene copies, the presence of different gene variants within a genome has been reported. Characterization of an organism by defining every single variant of tens to thousands of rDNA repeat units present in a eukaryotic genome would be quite unreasonable. Here we provide an alternative approach for the characterization of a set of internal transcribed spacer sequences found within every rDNA repeat unit by implementing direct sequencing methodology. The prominent allelic variants and their relative amounts characterizing an individual can be described by a single sequencing electropherogram of the mixed amplicon containing the variants present within the genome. We propose a method for rational analysis of heterogeneity of multicopy genes by compiling a profile based on quantification of different sequence variants of the internal transcribed spacers of the freshwater sponge *Ephydatia fluviatilis* as an example. In addition to using conventional substitution analysis, we have developed a mathematical method, the proportion model method, to quantify the relative amounts of allelic variants of different length using data from direct sequencing of the heterogeneous amplicon. This method is based on determining the expected signal intensity values (corresponding to peak heights from the sequencing electropherogram) by sequencing clones from the same or highly similar amplicon and comparing hypothesized combinations against the values obtained by direct sequencing of the heterogeneous amplicon. This method allowed to differentiate between all specimens analysed.

## Introduction

Ribosomal RNA genes (rDNA) have been widely used in taxonomy, biogeographic and phylogenetic analyses. A eukaryotic genome has tens to thousands of rDNA copies, containing genes for 18S, 5.8S, and 28S rRNAs. Between these genes, on either side of the 5.8S rRNA gene, the internal transcribed sequences (ITS), ITS1 and ITS2, are located [1]. In order to preserve the functionality of multicopy genes, concerted evolution is at play. However, homogenisation of all the gene variants is not always complete [2]. Divergent copies may interfere in obtaining adequate results from phylogenetic [3] or phylogeographic analysis [4]. Therefore it is crucial to distinguish between real informative allelic variants and the misleading single mutated copies, to achieve reproducible findings. For this reason, a small number of cloned sequences may not suffice to describe the whole complement of the genome and there is no guarantee that these few sequences are actually the most prominent and true descriptors for the individual studied. To overcome this problem, the allelic variants obtained should be quantified (either in the absolute or relative quantity they possess within the genome) in order to assess their importance (representability).

Previously described methods that employ PCR and direct sequencing for determining allele frequencies in pooled DNA (based on the peak heights of different nucleotides in the same position on the electropherogram) have been focused on analysing alleles that differ by nucleotide substitutions [5], [6]. Whereas, if the gene variants differ due to insertion or deletion events, and substitutions may be absent, a different strategy is needed to adequately analyse the heterogeneity of the gene pool. We hereby describe a method that also allows for the quantification of the amounts of different alleles containing heterogeneities caused by indel events. We use the ITS sequences of the freshwater sponge *Ephydatia fluviatilis* as an example, and describe intra-individual heterogeneity in this species for the first time. Our method enables to distinguish between individuals by compiling a specific profile for each individual analysed. Comparing these profiles can help us to assess which specimens are similar enough on a genetic level to be used together for one analysis e.g. for separating an enzyme of interest or compiling a cDNA library.

## Materials and Methods

### Sample collection and gemmule culture

Sponges containing gemmules were collected manually from shallow depths (up to 0.5 m) from 6 sites in Estonian rivers and streams from 3 drainage basins (see [7]) – the Peipsi-Pihkva Lake and the Narva River drainage basin: River Piusa (specimen Pi) and River Rõuge (specimen R); the Gulf of Finland drainage basin: Stream Vanamõisa (specimens V1, V2, V3, V4, V5 and V6); the Moonsund Sea and the Gulf of Riga drainage basin: River Lokuta (specimen L), River Pedetsi (specimens Pe1 and Pe2) and River Kõpu (specimens K1 and K2). The study was approved by The Estonian Environmental Board. No additional permits or approvals were required. The cosmopolitan sponge species *Ephydatia fluviatilis* is a representative of the phylogenetically lowest multicellular animals and there are no ethics regulations concerning this species. *E. fluviatilis* is widely distributed in Estonia and not under protection. The sites of collection were not located in protected or privately owned areas.

To identify the species by light microscopy, spicule preparations were made by treatment of aliquots of samples with concentrated nitric acid for 10 min at 95°C. After removing the organic matter the spicules were extensively washed with water and mounted on slides.

Gemmules were isolated from sponge specimens and stored at 4°C in mineral medium (M medium: 0.1 mM NaHCO_3_, 0.05 mM Na_2_SiO_3_, 0.01 mM KCl, 0.1 mM MgSO_4_, 0.2 mM CaCl_2_ [8]). In parallel,samples of sponge material were frozen in liquid nitrogen and stored at −20°C.

Before starting, the culture gemmules were treated with 1% hydrogen peroxide for 1 minute to minimize bacterial and fungal contamination [8]. Sponges were hatched on Petri dishes in M medium. Fully functional young sponges were removed from the dishes using a scraper and dispersed in calcium and magnesium free medium (CMF medium: 0.1 mM NaHCO_3_, 0.005 mM Na_2_SiO_3_, 0.01 mM KCl). Gemmule shells were discarded by a short sedimentation and the spicules were eliminated by filtration of the suspension through nylon gauze (50 µm mesh) [9]. The cells to be used for DNA extraction were isolated by centrifugation (500×g, 4°C, 10 min), frozen in liquid nitrogen and stored at −20°C.

Gemmules from specimens K1, K2, L, Pe1, Pe2, Pi, V1 and V2 were tested for their fusibility to distinguish the specimens belonging to the same strains [10]. Two gemmules from different samples were placed for hatching close enough on a Petri dish and the possible fusion of formed young sponges was monitored. The results were photographed using a Zeiss Axiovert 200 M microscope.

### DNA extraction

The sponge genomic DNA was extracted using CTAB technique [11] according to the protocol described earlier [7]. DNA extraction from gemmules was performed using the same technique. In the experiments where only 1 or 5 gemmules were used they were crushed with pipette tips in 50 µl M medium and the volumes of liquids used were ¼ of those described by Lopp et al. [7].

### PCR amplification, cloning and sequencing

The ITS region including ITS1, 5.8S gene and ITS2 was amplified using the primers 18Sfw: 5′-TAC ACA CCG CCC GTC GCT ACT A and 28S5′rev: 5′-GAC GTG CCT TTC CAG GTC AAC TT. The primers were designed to include the invariant sequence before the heterogenic positions in the amplicon. This allowed the assessment of the quality of the sequencing electropherogram data obtained.

The PCR amplifications were performed either in 30 µl or 50 µl reaction volume containing about 1, 10 or 100 ng genomic DNA and 0.5 µM of each primer, using 2× PCR Master Mix (Fermentas). The temperature profile was as follows: initial denaturation at 95°C for 1 min, followed by 30 or 35 cycles of denaturation at 95°C for 45 s, annealing at 55°C or 61°C for 45 s, and extension at 72°C for 1 min with a final extension at 72°C for 10 min.

The success of PCR amplifications was initially evaluated by electrophoresis of the products in 1% agarose gel. The amplification products were purified using the JetQuick PCR Purification Kit (Genomed, Germany).

DNA fragments of ∼1091 bp obtained by PCR amplification were directly sequenced using ABI PRISM 3130 Genetic Analyzer (Applied Biosystems) with the primers mentioned above. All sequencing PCR procedures were conducted using ¼ of the recommended amounts of reagents.

The PCR products from five specimens were cloned into the pTZ57R/T vector using InsTAclone PCR Cloning Kit™ (Fermentas), following the manufacturer's instructions. Either vector specific primers or PCR product specific primers were used to sequence the cloned fragments.

### Data analysis and the proportion model

The data obtained by sequencing were analysed using the BioEdit program (http://www.mbio.ncsu.edu/bioedit/bioedit.html). Only sequencing data that displayed electropherograms of good quality were used.

The absence or presence of notable intraindividual heterogeneity was assessed by visual examination of the electropherograms obtained by direct sequencing. The presence of heterogeneities was indicated by double peaks in substitution positions, and by a series of mixed peaks in case of indel events, both positioned after a sequence of good quality. In the case of nucleotide substitutions, the peak heights corresponding to the particular nucleotides in the same position on the electropherogram were compared as previously described [12]. A novel modelling strategy, the proportion model method, was developed for relative quantification of the alleles differing due to indels. This method allows for quantifying the sequences having insertions or deletions within a pool of otherwise highly similar sequences.

The method is based on comparing the signal intensity values obtained by direct sequencing of the amplicon to the corresponding intrinsic values. The intrinsic intensity values for each nucleotide in the sequence are used to model the same sequence being placed in different frames in various proportions.

Each nucleotide has its specific intensity value in each position because the intensity measured for each nucleotide depends on the nucleotides that precede it in the sequence. To assemble the model, the information obtained by sequencing 48 clones was used for finding the intrinsic (expected) intensity values for each mainly represented nucleotide in every position of the alignment. The numerical values were acquired by exporting trace values from BioEdit and extracting only the reads that corresponded to the points where the program had placed the nucleotides.

As each run has its specific signal intensity level, the signals from different runs were normalized, so that the mean of the intensities in a sequence would be as close as possible to 1000 units. Then to obtain the expected values, the mean value among clones for a particular nucleotide in each position was found. The normalization was needed also because different primers were used for sequencing the clones, so any particular nucleotide in the rDNA was positioned at a different distance from the primer.

The data obtained by direct sequencing were normalized in a similar manner, but because the maxima of the minor peaks did not always coincide with the points where the nucleotides were placed by the program, the greatest intensity values registered for other nucleotides for each position in a 5 point window from these data were also found. To take into account all nucleotides registered at the position, these intensity values of a position were summed up, and the sums were used for normalization.

The intrinsic values were used to model the expected signal intensities for different frameshift (or phase shift [13]) combinations, depicting the mixture containing various amounts of sequences of different length due to indel events in the specific region. Signal intensities from these simulations of hypothetical combinations were compared to the information obtained by direct sequencing and the best fitting situation was established; this defined the relative contents of particular length variants in the amplicon. The part of the sequence that was used for comparison comprised of at least 200 nt following an indel position.

Since we found different annotations in NCBI GenBank for the borders of *E. fluviatilis* ITS sequences, we aimed to avoid further confusion about the lengths of ITS sequences described and designated the borders as follows: the last 8 nucleotides of 18S are GATCATTA and the first of 5.8S ACAACTTC; between those sequences the ITS1 is positioned. ITS2 is positioned between the end of 5.8S (TCTGAGCG) and the beginning of 28S (CGCTGAAT).

### Data Access

The sequences of the clones have been submitted to the NCBI GenBank (http://www.ncbi.nlm.nih.gov/) under accession numbers KC243990–KC244049.

## Results

### 1. Elaboration of the method

**Figure 1:**
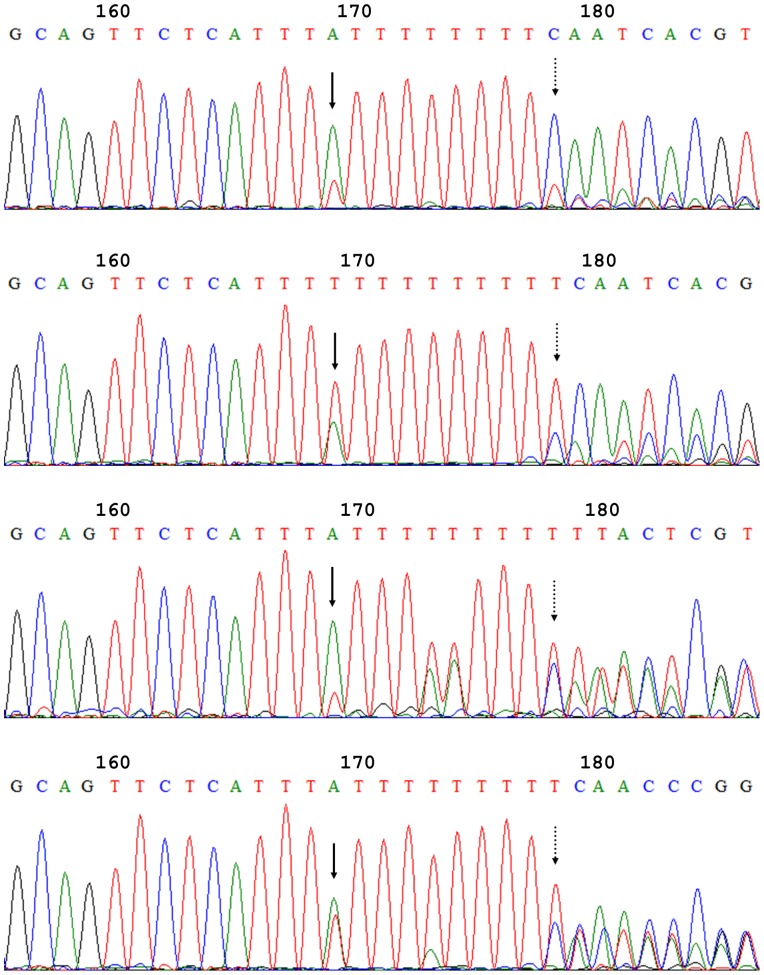
Examples of results from direct sequencing of ITS1. Aligned fragments of sequence electropherograms (sequenced using the forward primer) starting from Position 156 of ITS1. Samples (from top to bottom): V1, V2, L, and K2. Black arrows indicate the substitution in Position 169; dashed arrows indicate the first position where a deletion has occurred in more than 5% of sequences in a sample (an indel).

**Figure 2:**
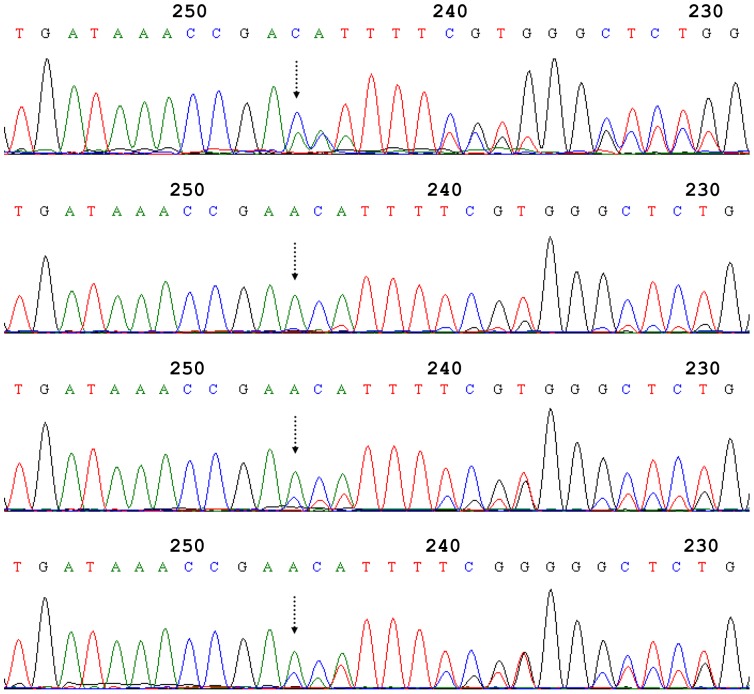
Examples of results from direct sequencing of ITS2. Aligned fragments of sequence electropherograms (sequenced using the reverse primer). Samples (from top to bottom): Pe2, R, K2, and V2. Dashed arrows indicate the position of an indel.

The amplicons obtained from all 13 *Ephydatia fluviatilis* specimens analysed contained the ITS region, including the 3′ end of the 18S gene, the full length ITS1, 5.8S gene, ITS2, and the 5′ end of the 28S gene. Direct sequencing of those amplicons displayed intra-individual heterogeneities in all specimens analysed. The examples of heterogeneities revealed by direct sequencing are displayed in Figures 1 and 2 for ITS1 and ITS2, respectively. The direct sequencing electropherograms obtained for a single individual exhibited significant similarity, whereas the similarity decreased noticeably in the case of different individuals.

**Figure 3:**
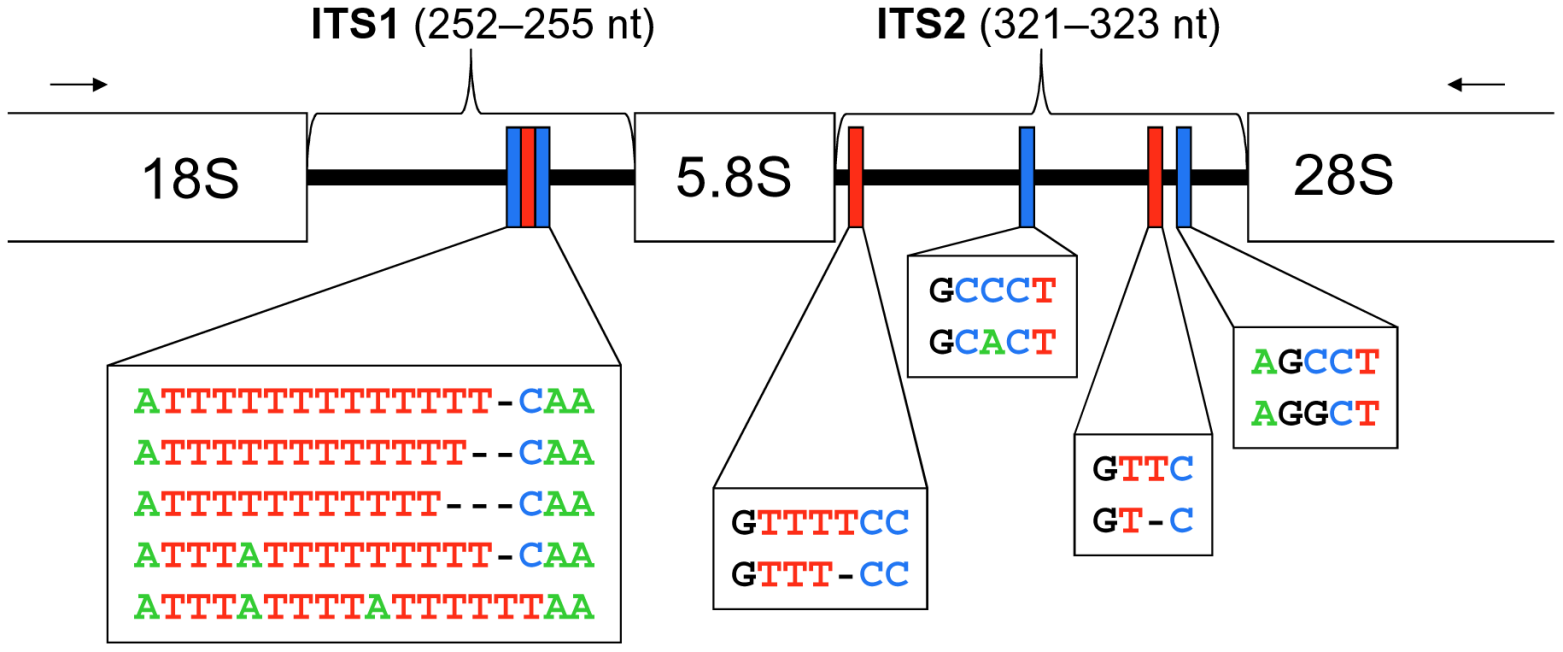
Schematic representation of heterogeneities found in ITS1 and ITS2 revealed by cloning from 5 specimens. Red and blue rectangles mark the locations of indels and substitutions, respectively. The heterogenous positions (accompanied by a few surrounding nucleotides) found in those locations are given in the boxes below.

To elucidate the visible heterogeneity, the amplicons from the specimens K1, Pe1, Pe2, R, and V2 were cloned and 12 clones per specimen were sequenced. The summary of the heterogeneities in the ITS region displayed by the clones is depicted on Figure 3 (only heterogeneities that were visible in more than one clone from a specimen are shown). Some of these sequences were specific to one animal; others were represented in several animals. All individual sequences obtained from clones are posted in NCBI GenBank (http://www.ncbi.nlm.nih.gov/) under accession numbers KC243990–KC244049.

The data obtained by sequencing individual clones suggested considerable repeatability of signal intensity values; the mean standard deviation of the standardized signal intensities for each position was around 5%. Every position in the sequence had a specific repeatable value for a particular nucleotide. It allowed us to compare the results of different sequencing experiments of highly similar sequences numerically and to construct the proportion model (described in Materials and Methods) and perform overall quantitative analysis of the complex electropherogram obtained by direct sequencing. In order to quantify the rate of substitution in a position more accurately, the expected signal intensity values were taken into account for the two nucleotides appearing in the position analysed. Besides substitutions, our proportion model permitted us to unravel the contribution of indels to the complex electropherograms obtained by direct sequencing. This makes our method advantageous for characterizing a sponge individual based on direct sequencing the ITS region.

### 2. Repeatability and robustness of the method

#### 2.1. Repeatability of the results obtained using the proportion model

The first experiments to test the repeatability of the method were performed using a single DNA extraction from young sponges grown *in vitro* from about a thousand gemmules of the specimen V2 as an example. The relative amounts of the ITS1 sequence variants of different length and variants containing the substitution in Position 169, found in these different PCR experiments are presented in Table 1. The results from the substitution analysis are rounded to integer percentage values, because when analysing a substitution, information only from one position in the sequence is used (the peak heights from two peaks in the same position in case of a single nucleotide substitution), while in the length analysis using the proportion model, hundreds of downstream positions following the site of an indel provide the data, and therefore a more accurate assessment can be made.

**Table 1:** Percentages of ITS1 sequences of different length and nucleotides found in ITS1 Position 169 in different PCR conditions(the same DNA extract from the sponge specimen V2 was used in all amplifications).

PCR conditions	ITS1 length (nt)		Nucleotide inITS1 Position 169	
	253	254	A	T
1) 100 ng of DNA	25.49	74.51	34	66
2) 100 ng of DNA	24.76	75.24	35	65
3) 100 ng of DNA	24.84	75.16	36	64
Primers 2:1	25.41	74.59	34	66
Primers 1:2	24.68	75.32	37	63
10 ng of DNA	26.17	73.83	36	64
1 ng of DNA	27.29	72.71	38	62

The results from three parallel PCR amplifications with 100 ng of DNA from the same extraction, conducted in same conditions, showed a variance of less than 1% for the ITS1 length analysis (Table 1), which is far less than the permitted error rate of 5% (established from clone sequencing results), and a variance of 2% for the substitution analysis, which is also significantly lower than the permitted error rate of 10%.

#### 2.2. Robustness of the method

To assess the robustness of the method, first the concentrations of either primer in the PCR mixture were doubled (Table 1). This had no influence on the results: the absolute deviation from the mean value of 100 ng experiments was from 0.35% to 0.38% for the length analysis.

Secondly, the amounts of template DNA were varied. The reactions conducted with 10 or 1 ng of DNA were more variable than those with 100 ng, the absolute deviations from the mean values of 100 ng experiments being 1.14% and 2.26%, respectively (Table 1); showing that the amount of template DNA can be reduced to 1 ng with the experiment still giving reliable results.

Further on we found that the method was sensitive to limiting the amount of starting sponge material, e. g. number of gemmules. When extracting the DNA from just one gemmule or five gemmules of the same specimen V2, the yield of DNA was minute (about 34 ng DNA for 1 gemmule, 240 ng to 500 ng for 5 gemmules, vs ∼10 000 ng for young sponges grown from about a thousand gemmules; for PCR amplifications ∼1 ng, 8 ng and 17 ng vs 100 ng of DNA was used, respectively), and therefore the accuracy of the results of corresponding PCR amplifications were comparable to those where 1 ng or 10 ng of DNA extracted from young sponges had been used.

In the experiments where DNA was extracted from young sponges grown from about a thousand gemmules, only the ITS1 sequence variants of 253 nt and 254 nt were detected above the 5% threshold (established from acceptable noise level in sequencing electropherograms). Whereas amplifying the DNA extracted either from 1 gemmule or 5 gemmules, sequence variants of 252 nt and 255 nt length also became detectable above the 5% threshold (in experiments with 100 ng of DNA used in PCR they were detected around 3% and 2%, respectively, since the noise level in the particular electropherograms was very low; for an example see Figure 1, second electropherogram from the top). Mostly these variants were represented in quantities of less than 10%. The accuracy of the results remaining close to the detection threshold may be affected by possible distortions from random noise in sequencing data; therefore the results for the most represented variants are more reliable. Since a greater accuracy cannot be guaranteed, the following results are rounded to integer percentage values.

In detail, the experiments with 5 gemmules, sequence variants of 252 nt ITS1 length were represented by 7% (17 ng DNA in PCR) and 5% (8 ng DNA in PCR); for 1 gemmule (1 ng DNA in PCR) this value was 5%. For 5 gemmules, the contribution of sequence variants of 255 nt ITS1 length fell below 5% in one case (for the PCR with 17 ng DNA) and was therefore considered equal to 0% but in a parallel experiment (for the 8 ng DNA PCR) the value was 8%. For 1 gemmule (1 ng DNA in PCR) the amplified sequence variant 255 nt ITS1 comprised 34% of all variants. This discrepancy beyond permissible variance indicates that the method is sensitive to using very small quantities of starting material, i.e. less than 5 gemmules, for DNA extraction.

The results for more frequently represented sequence variants of 253 nt and 254 nt long ITS1 were also more variable in the case of a small numbers of gemmules used as starting material, but the discrepancies were not as drastic as in the case of 252 nt and 255 nt variants. The 253 nt sequence variants comprised 29% and 25% in 5 gemmules experiments and 20% for 1 gemmule (compared to 25% which was considered to be descriptive of that specimen by the experiments where 100 ng of DNA was used for PCR). For 254 nt sequences, the respective values were 63%, 62%, and 41% (compared to 75%, respectively). These results suggest that amplification of DNA isolated from too small amounts of starting material (fewer than 5 gemmules) cannot guarantee accurate results. To ensure the presence of sufficient amount of template DNA for quantitative analysis all further experiments were conducted using 100 ng DNA in a PCR.

The proportions of heterogeneities in ITS1 found using the direct sequencing data were comparable to the amounts of sequence variants from 12 clones of the same specimen V2. When analysing the substitution in ITS1 Position 169, the results were very similar: 4 clones had A in that position and 8 clones had T. In case of the length analysis the results were not that correlated: the majority of clones, 8 of them, had an ITS1 length of 254 nt, 2 clones had 253 nt, but 2 clones had the shortest ITS1 variant, 252 nt, that was represented by less than 5% in the direct sequencing results (when 100 ng of DNA was used for PCR).

**Figure 4:**
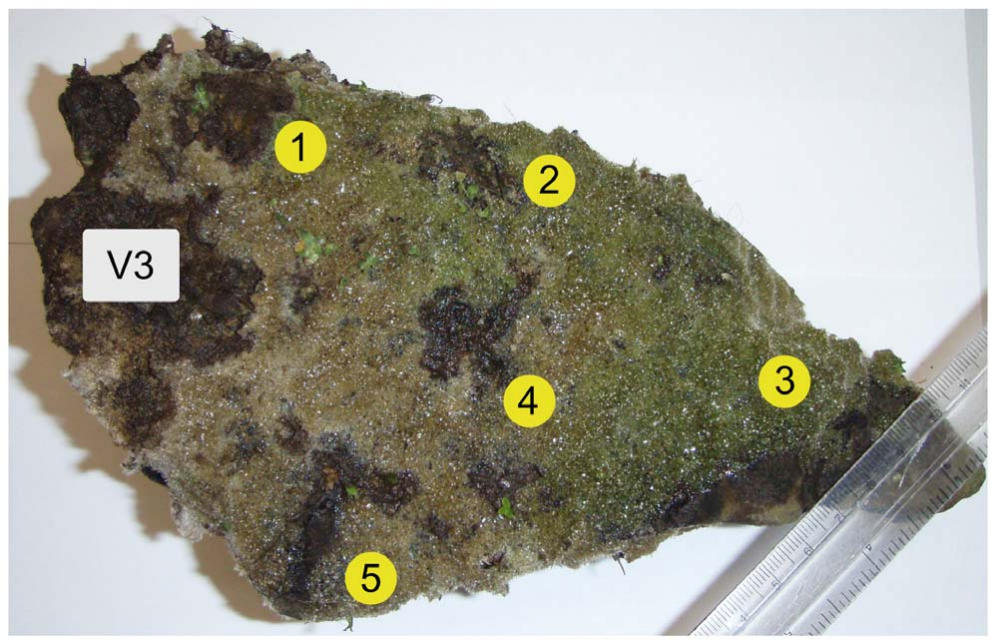
Sites of samples taken from a single individual (specimen V3). The yellow labels indicate the sites of the sponge where the samples were taken. The excised sponge areas were always smaller than the label area.

The results for the samples taken from various parts of an adult sponge (specimen V3; Figure 4) displayed a uniform distribution of the amounts of all heterogeneities found in ITS1 (see Table 2). The highest variability was noted for the most represented length variant (254 nt): the maximum range between samples 1, 2, 4, and 5 was 3%, which is still smaller than the permitted error rate of 5%. In the case of Sample 3 the electropherogram was not of good quality as the noise level exceeded 10% of the average signal intensity, and therefore the sequencing results were not eligible for the quantitative analysis (the direct sequencing electropherograms are shown in File S1). However, including the data of Sample 3 into the analysis would only increase the maximum range for 254 nt length variant to 6%. There were no substitution positions found in either ITS1 or ITS2 sequences for this specimen. Most of the ITS2 sequences obtained for this specimen were of such bad quality, that they were unsuitable for precise analysis. However, visual examination of the chromatograms confirmed that in all of them the length variant of 323 nt was dominant, and the other ones, if present, were there in indistinguishable amounts or comparable to the noise level.

**Table 2:** Percentages of ITS1 sequences of different length contained within samples taken from different sites of the same specimen, V3.

V3: Sample	ITS1 length (nt)			
	252	253	254	255
1	6	31	57	5
2	7	30	56	6
3*	*10	*31	*51	*8
4	8	32	54	6
5	8	31	55	6

*sequencing results were unsuitable for the analysis as the noise level on the electropherogram exceeded the permissible 10%.

### 3. rDNA heterogeneities displayed in Estonian *E. fluviatilis* population

All 13 specimens of *E. fluviatilis* studied displayed intraindividual heterogeneity in their ITS region (for direct sequencing electropherograms see File S1). According to the results of direct sequencing neither substitutions nor indels were detected in the sequence of 5.8S gene, the latter was identical to the sequences for *E. fluviatilis* disclosed in NCBI GenBank (accession numbers: AJ705048; EF151942; EF151951).

#### 3.1. Heterogeneities in the ITS1 region

Sequencing the clones of the ITS region amplified from 5 specimens (K1, Pe1, Pe2, R and V2) revealed 6 informative positions in ITS1 that were confirmed by the results of direct sequencing, three of them being substitution positions and others being the sites of indels: the length of ITS1 varied from 252 nt to 255 nt. The direct sequencing of the amplicons from other specimens showed additional heterogeneities in ITS1. For example, in Position 173 two specimens (L and K2; see Figure 1, two lower electropherograms, respectively) displayed a distinct signal for A in addition to T. The same substitution also appeared in one of the clones from the specimen R, whereas direct sequencing of the ITS1 region amplified from the same specimen showed the signal intensity caused by this substitution below the 10% threshold. Accordingly, this substitution was not considered as one of those describing the particular animal.

The most heterogeneous substitution position in ITS1 was 169, where all the previously published sequences had T. The majority of specimens analysed in this study, except V3, had both kinds of sequence variants, i.e. having either A or T in this position, in different proportions; the specimen R displayed exclusively A.

All heterogeneities in ITS1 appeared between positions 163 and 180. Five of them were substitutions: 163 G/T, 169 A/T, 173 A/T, 174 A/T, 180 T/C; all given in relation to the consensus of different *E. fluviatilis* sequences found in NCBI GenBank, numbers representing the positions in ITS1. The ITS1 length variation was caused by the differences in a homopolymer T-track length with indels in positions 177 to 179. The substitution rates, as well as relative amounts of ITS1 variants of different length, for all specimens analysed are presented in Table 3. Taken together, these data make up the ITS1 profiles for all 13 sponge individuals studied.

**Table 3:** ITS1 heterogeneities in different individuals: percentages of ITS1 sequences of different length and of nucleotides in substitution positions analysed.

Sample	ITS1 length (nt)				Substitution in ITS1				
					163	169	173	174	180
	252	253	254	255	G (T)	A (T)	A (T)	A (T)	T (C)
K1	0	23	77	0	0	52	0	0	0
K2	0	34	34	32	0	63	17	0	0
L	0	42	5	52	0	83	43	48	43
Pe1	0	6	35	59	0	74	0	49	41
Pe2	0	6	32	62	0	75	0	54	41
Pi	0	12	33	56	0	68	0	0	0
R	0	7	88	5	0	97	0	0	0
V1	0	82	10	8	0	79	0	0	0
V2	0	25	75	0	0	35	0	0	0
V3	7	31	56	6	0	0	0	0	0
V4	0	19	81	0	0	48	0	0	0
V5	0	18	82	0	0	61	0	0	0
V6	0	19	81	0	17*	63	0	0	0

The nucleotides in brackets are the ones most frequently represented in sequences found from NCBI GenBank.

The asterisk (*) marks the substitution that was not found in any of the clones and therefore is calculated using direct sequencing results only.

For specimen V2 the results displayed here are the average from the experiments where 100 ng of DNA was used; for specimen V3 they are the average from four successful experiments (see Table 2).

#### 3.2. Heterogeneities displayed in ITS2

Heterogeneities detected in ITS2 were more dispersed. Substitutions occurred in ITS2 positions 151 (C→A), 244 (T→A) and 268 (C→G) (Table 4). Our proportion model allowed us to detect single substitutions even in the case where they were located in the sequence following indel positions, where the sequence appeared as a mixture due to the different allelic variants being visible in different frames in the electropherogram. One of those substitutions discovered with our model appeared in Position 244 and occurred in two specimens, V1 and K2; neither of the specimens was subjected to cloning.

**Table 4:** ITS2 heterogeneities in different individuals: percentages of ITS2 sequences of different length and those of nucleotides in the analysed substitution positions.

Sample	ITS2 length (nt)			Substitution in ITS2		
	321	322	323	151*	244*	268
				A (C)	A (T)	G (C)
K1	32	29	39	51	0	0
K2	21	62	17	17	28	0
Pe1	54	19	27	0	0	0
Pe2	48	36	13	0	0	0
Pi	0	70	29	0	0	0
R	10	73	17	0	0	32
V1	87	8	5	0	86	0
V2	20	25	55	0	0	0
V3	5	7	88	0	0	0
V5	0	8	92	0	0	0
V6	0	7	93	0	0	0

The substitution results that are calculated from sequencing data of mixed sequences (the area following indels) are marked with an asterisk (*).

The indel events in ITS2 had occurred in Positions 13 and 247. Both of them involved tracks of T nucleotides, the first one starting from Position 10 (variants displayed were TTTT and TTT) and the other one in Position 246 of ITS2 (being either TT or T). These variants occurred in different combinations. The amounts of sequence variants of different ITS2 length are given in Table 4.

#### 3.3. ITS profiles of sponge strains

**Figure 5:**
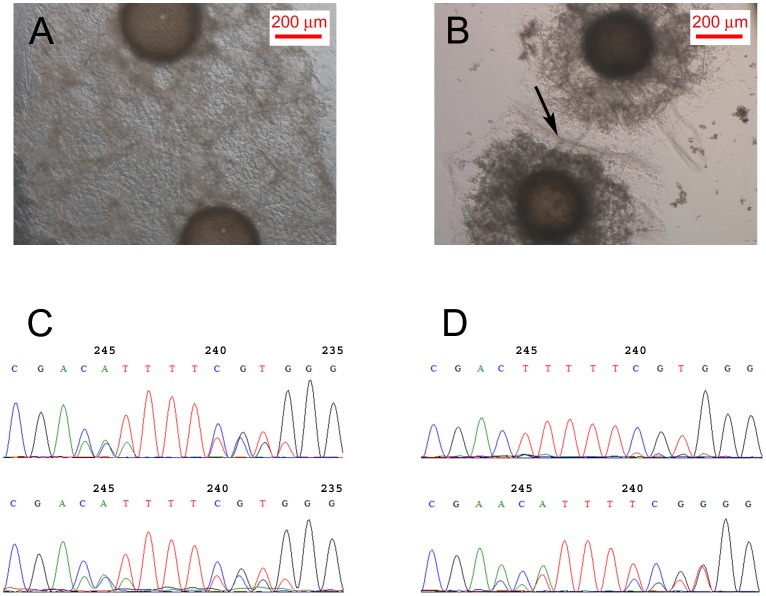
Examples of results from fusibility experiments. Young sponges grown from gemmules of the same strain, Pe1 and Pe2 (A) and of different strains, V1 and V2 (B) and aligned fragments of sequence electropherograms (c and d, respectively) from direct sequencing with the reverse primer 28S5′rev (around the region of the indel at position 247 of ITS2). The black arrow (B) shows the barrier formed between two individuals that signifies them belonging to different strains.

Specimens collected from one site of River Pedetsi, Pe1 and Pe2 belonged to the same strain (according to the definition by Van de Vyver 1970): young sponges grown from gemmules of those two specimens fused completely and formed a single functional animal (Figure 5A). All other specimens tested for fusibility formed a barrier between two young individuals when grown pairwise from gemmules, i.e. they belonged to different strains. An example of two specimens of different strains found from one locality, the stream Vanamõisa, is the one involving V1 and V2 (Figure 5B).

The ITS profiles of the specimens belonging either to the same strain or to different strains were compared, exemplified by the aforementioned specimens. Figure 5 (C and D) shows aligned fragments of sequence electropherograms from direct sequencing with the reverse primer 28S5′rev (around the region of the indel at Position 247 of ITS2) of the respective specimens, below the images of the young sponges grown from gemmules of those specimens. The full ITS profiles of the specimens are enclosed in Tables 5 and 6. These data refer to highly similar ITS profiles for the specimens of the same strain, while the specimens of different strains had distinctly different ITS profiles.

**Table 5:** Comparison of ITS1 profiles of specimens of the same strain and of different strains.

Sample	ITS1 length (nt)				Substitution in ITS1				
					169	173	174	180	163
	252	253	254	255	A (T)	A (T)	A (T)	T (C)	G (T)
Pe1	0	6	35	59	74	0	49	41	0
Pe2	0	6	32	62	75	0	54	41	0
V1	0	82	10	8	79	0	0	0	0
V2	0	25	75	0	35	0	0	0	0

**Table 6:** Comparison of ITS2 profiles of specimens of the same strain and of different strains.

Sample	Substitution in ITS2			ITS2 length (nt)			Deletion in ITS2	
	151	244	268	321	322	323	13	247
	A (C)	A (T)	G (C)				-	-
Pe1	0	0	0	54	19	27	65**	62
Pe2	0	0	0	48	38	13	70**	64
V1	0	86*	0	87	8	5	95**	87
V2	0	0	0	20	25	55	32**	33

An asterisk (*) marks the substitution results calculated from sequencing data of mixed sequences (the region following an indel).

Two asterisks (**) mark the results calculated using other results of length analysis obtained using the proportion model.

## Discussion

The method developed in this study allows for the analysis of a mixture of amplicons derived from a pool of highly similar sequences (e.g. multicopy genes) by direct sequencing. In addition to the conventional substitution analysis, our method considers the contribution of indels into the sequence pattern of a multicopy gene of an individual. For the first time, intraindividual heterogeneity of *E. fluviatilis* is described, and a distinctive ITS profile of each individual analysed is composed.

The direct sequencing approach proved suitable for assessing the informativeness of the heterogeneities found in the clones because the data from direct sequencing proved to be repeatable. As a prerequisite for this method, cloning the amplicons and subsequent sequencing of a small number of clones is still required to establish the intrinsic signal intensity values. Using the intrinsic values is a novel way to quantify indels but it also allows us to assess the amounts of sequence variants containing certain substitutions with more accuracy than possible with using only the data from the electropherogram of direct sequencing.

In this work we used the direct sequencing approach together with the proportion model for both the ITS1 and ITS2 regions, treating them separately. Although the sequence variants of the whole ITS region cannot be presented using this approach, the results provide specific profiles for every individual analysed. Even though the results of direct sequencing may not describe the actual and entire set of sequence variants (i.e. which combinations of heterogeneities exist), these results are reproducible; accordingly the method is applicable. The sequences obtainable from clones of the mixed amplicon cannot depict the existing heterogeneities better, as the same PCR technique with all its errors is used.

The proportion model method is used with maximal accuracy if indels are positioned with sufficient intervals in the sequence under investigation. These distances can be determined from the cloned sequences. The fact that the positions analysed in this study were more than 200 nt apart meant that the proportion model was suitable. Whenever the distance between indels is just a few nucleotides, the results may be less precise, because the variation of single intensity values will have more influence on the results if data from only a few positions can be used. Also, the noise level is higher in the sequence following additional indels since individual signals are weaker because the signal is spread out to more positions. High noise level in sequencing data can affect the results by artificially increasing the content of heterogeneities that are present in relatively small amounts. Therefore it should be kept in mind while comparing profiles of individuals that for drawing any conclusions, the amounts of the most represented heterogeneities are more reliable than those of the ones closer to the detection limit.

The method has its limitations in the amount of starting material. Reducing the amount of template DNA may lead to more variable results due to random events (like PCR drift described by Wagner et al. [14]). Different PCR results can be caused by particular sequence variant(s) having advantages in being replicated; even if this advantage is small, it can amount to a significant difference [14]. In the amplification of different ITS sequence variants, the amount of which is sufficient for detection, the PCR conditions play a crucial role [15].

The smallest amount of DNA, 1 ng, used in our experiments, was sufficient to obtain equivalent results to 100 ng from the same extraction (from young sponges grown from about a thousand gemmules of the same sample). On the contrary, 1 ng of DNA extracted from a single gemmule, may not contain an ample amount of template DNA to give representative results. A single gemmule displayed different ITS1 length variant contents compared to the other experiments of the same sample (the shift was around 34% for 254/255 nt). DNA extracted from five gemmules of the same sample showed less, but still detectable inconsistency with young sponges grown from about a thousand gemmules (see 2.2. Robustness of the method). Nevertheless, in practice there is much more available material for characterization of an individual and the analysis of a single gemmule is neither necessary nor practical.

There have been only a few studies describing intraindividual heterogeneity in sponges. In the first publication [16], the ITS region of the marine sponge *Crambe crambe* was analysed. The authors reported that all intraindividual heterogeneities displayed by the clones sequenced were also visible on the electropherogram of the amplicon sequenced directly after PCR. Wörheide et al. [17] also performed direct sequencing of ITS regions of several marine sponges and compared the results with sequences from clones to compare the intraindividual heterogeneity appearing. Most of the results of that study were based on heterogeneities found by sequencing clones, including those missing on the electropherograms of direct sequencing. Generally, the analysis of electropherograms does not seem to be a common practice and often the ambiguities may be overlooked. A recent example is the study of the ITS2 sequences from the elephant ear sponge *Ianthella basta* [18]. It is difficult to decide whether the representative set of sequence variants was revealed in that study, as the authors do not specify the limiting amount of the minor nucleotide present (the peak heights on the electropherogram) in the direct sequencing results at a position considered to be a heterogeneous one.

Data published on the heterogeneity of the ITS sequences of *E. fluviatilis* have been scarce. Itskovic et al. [19] found that the ITS1 fragments from two *E. fluviatilis* individuals from Japan were of a length of 254 nt. All specimens collected from Estonia contained this length variant (it was the main variant in about half of the samples) among others; up to 4 different length variants in a single sample (252 nt to 255 nt) were found, and there were no cases where only a single ITS1 length variant was present. Compared to our sequences the two sequences from Japan displayed a few additional indel sites and around ten substitution positions setting them apart from each other and from our sequences.

For ITS2, the existence of different length variants in *E. fluviatilis* has been found in individuals separated by a long geographical distance: from Italy [20], the length variant of 324 nt (323 nt by our annotation) and from Japan [19], the length variants of 321 and 322 nt by our annotation (289 nt by their own annotation). The specimens collected from Estonia had their ITS2 lengths from 321 to 323 nt. Both of the specimens from Japan contained the same deletions we had detected and 3 additional indel and 4 substitution positions. Gigliarelli et al. [20] described one substitution in ITS2, Position 263 (showing both A and G); by our annotation this position is 24^th^ in the beginning of 28S RNA gene. The authors did not specify the number of clones with either of those substitutions. Otherwise the two sequences from Italy were identical to one of the most popular variants we found in our clones. In the present study three informative substitution positions in ITS2 were found.

Freshwater sponges generally exhibit more conservancy in their ITS1 region than in ITS2. The middle parts of both regions are most conserved: 90% of sponges studied by Itskovic et al. [19] (16 accessions of Spongillidae, *Echinospongilla brichardi* and 2 Lubomirskiidae, *Baikalospongia bacillifera* and *Lubomirskia baicalensis*)) had 120 nt conserved in the middle part of ITS2 and 145 nt in the middle part of ITS1. This conservancy may result from the functionality of these regions. The heterogeneities found for *E. fluviatilis* in our study were also positioned towards the ends of the sequences. The variable portion of ITS1 spanned from Positions 169 to 180, the whole length of ITS1 being 252 nt to 255 nt. The indels in ITS2 were located even closer to the ends – in Positions 13 and 247 of ITS2, while the substitution positions were more dispersed along the region – in Positions 151, 244, and 268 (the ITS2 length ranged from 321 nt to 323 nt).

All length heterogeneities detected in the present study were caused by indels in tracks containing a different number of T nucleotides. Wörheide et al. [17] also described indels in homopolymer T repeats for 3 marine sponge species. The authors considered these sequences to be questionable because of possible polymerase slippage in the homopolymer region during the sequencing reaction. In our study the homopolymer track was between 1 to 13 nt, that is far below the limit (25 nt) suggested by the manufacturer of the sequencing kit [21]. The examination of sequencing electropherograms of the clones showed that only a few clones containing the longest homopolymer tracks displayed some noise after the repeat region.

The direct sequencing did not reveal any heterogeneity in 5.8S gene; its sequence was identical to the *E. fluviatilis* sequences disclosed in NCBI GenBank. A few inconsistencies were found in the clones sequenced; these can be attributed either to PCR or cloning artefacts or to pseudogenes, remaining under the detection limit for direct sequencing. A similar amount of noninformative heterogeneities were observed all over the sequence analysed. The fact that the ITS sequence variants found were represented in various proportions in different individuals – there was no single (or even a few) dominant variant(s) throughout the species – implies that a majority of these ITS sequence variants found do not belong to pseudogenes.

Every animal studied displayed an array of different sequence variants. rDNA repeats in *E. fluviatilis* have been mapped to two chromosomes [22]. Our data indicate that the homogenization of copies within chromosomes is far from being complete. Similarly, in humans the existence of up to three different IGS (intergenic spacer) classes in one rDNA block has been recorded [23]. rDNA has been shown to be positioned in clusters, which may contain copies in different orientation [24]; the clusters being positioned in different orientation may contribute to the incomplete homogenisation.

The number of different sequence variants in an individual's genome is very difficult to assess because the heterogeneities were present in highly varied proportions throughout the samples, and in various combinations in the clones sequenced. Furthermore, the copy number of rDNA sequences in the genome of *E. fluviatilis* is not known. The closest assessment that can be made is for another sponge, *Amphimedon queenslandica*, whose genome has been sequenced. *A. queenslandica* is estimated to have approximately 14.5 copies of rDNA sequences per haploid complement [25], (Reintamm, unpublished). The fact that we detected various heterogeneities in very different amounts across the specimens implies that the rDNA copy number for *E. fluviatilis* may be larger. Making it even more complicated, individuals of the same species can have very different numbers of rDNA copies because the clusters display both meiotic rearrangements and somatic mosaicism. It has been shown that in humans for example, the number of rDNA sequences even within a single cluster can vary in an enormous extent, from one repeat unit up to >140 repeats [26].

In our study we observed the discrepancy of the results between experiments with one gemmule and/or five gemmules and from those with the young sponges grown from about a thousand gemmules. It is possible that different sequence variants can appear in different relative amounts, and minor variants that could be ignored in the whole sponge sample, may seem to be dominating in a gemmule. It can also be proposed that single gemmules do harbour ITS variants in proportions different from the parent sponge. Intra-individual heterogeneity in sponges may be present either due to the heterogeneity within the genome or the chimerism of the animal [27]. Working with tiny amounts of material (DNA extracted from one larva or 1–2 mm^2^ of sponges body area), Blanquer and Uriz [27] demonstrated genetic intraindividual heterogeneity of a sponge, as well as genetic variation of its progeny. Unfortunately the repeatability of such experiments cannot be assessed, since the sponge material for DNA extraction is used up in a single experiment in such case.

Although our study of different parts of the whole sponge body of a single individual suggested that the animal was not mosaic concerning the ITS variants, the possibility exists that somatic mutations may have occurred, as described by Blanquer and Uriz [27]. The change of the ratios of sequence variants can also be caused by the frequent recombination of rDNA [28]. A large part of the cells in a gemmule might have been the decendants of a single cell where such DNA rearrangements had taken place.

It is also possible that chimerism in sponges is not observed while analysing a large amount of sponge material, because the cells are constantly moving around within the body of a sponge [29]. The cells containing different ITS sets may spread out more or less evenly all around the body and give the same results, if a sample of a sufficient size is taken from any part of the sponge body. However, in a gemmule the cells are fixed within its limits, and it is not known whether the cells there have aggregated from different parts of the body, or are the descendants of only some of the local cells. In the latter case, a single mutation in one cell may contribute (through a large population of its descendants) to obtaining different results for a gemmule compared to the parent sponge.

Taking it ever further, these results obtained with tiny amounts of the starting material can be considered as coming from different individuals, since a functional sponge can grow from a single gemmule, and the cells exiting from gemmules originating from one individual (or individuals of the same strain) can also form a single animal when the gemmules germinate in close proximity. Even though, concerning sponges, the issue of individuality has been debated for quite some time, most scientists have agreed to consider a morphologically separate sponge as an individual [30].

The different result for one gemmule might have arisen from not having an ample amount of copies of the genome in PCR for quantitative analysis. Considering that one gemmule contains around 500 binucleated cells [31], and the size of *E. fluviatilis* haploid genome has been estimated to be 0.37×10^9^ bp [22], a single gemmule should contain around 0.77 ng genomic DNA. Recent studies suggest that the gemmule of *E. fluviatilis* may contain thousands of cells [32]. Still, the amount of DNA in a single gemmule would remain in the range of a few nanograms. However, in the present study the amount of DNA extracted from one gemmule appeared to be around 34 ng referring to the content of other components besides sponge DNA, absorbing at 260 nm.

Even though the amount of DNA from commensals is minimized by analysing gemmules and young sponges grown *in vitro* from gemmules instead of adult sponges collected from natural habitats, there may still be bacterial DNA present in the extraction. Besides containing sponge cells, gemmules have been shown to house bacteria and also eukaryotic cells, most likely symbiotic ones [33]. In sponges, vertical transmission of symbionts occurs; the presence of symbionts in larvae has been recorded for several species [34], [35], [36], [37]. Sequencing the genomes of *A. queenslandica* larvae also yielded many bacterial sequences [25]. It would not be surprising to find the specific bacterial community of *E. fluviatilis* [38] in gemmules, since this would serve to conserve the same community in their adults. Presumably, the presence of DNA from other organisms did not affect the results of our analysis, as the primers were designed specifically for freshwater sponges and the sequences obtained were explicit for *E. fluviatilis*.

We can further propose that besides individuals of *E. fluviatilis* also the strains of *E. fluviatilis* can be distinguished by using our method. The strains are determined by immunological mechanisms underlying recognition of their own and foreign cells. For different sponges to be able to form a single unified body and their cells to adhere, the aggregation factors (supramolecular proteoglycan-like complexes) and their interactions with cell surface receptors must be specific enough [39], [40], therefore the cells need to be similar enough on a genetic level as well. These recognition mechanisms are not directly linked with rDNA, nevertheless studying the ITS profiles allows us to assess the genetic similarity of individuals.

In our study the ITS profiles of the individuals of the same strain (specimens Pe1 and Pe2) were recognizably similar; the only difference was in ITS2 length variants which appeared in different proportions (Figure 5, Tables 5 and 6). The two samples from Vanamõisa location belonging to different strains (samples V1 and V2) exhibited 10 distinguishing features in their ITS profiles (Tables 5 and 6). Also, all the other specimens, proven to belong to different strains, had features setting them apart from all others. To confirm that the specimens do belong to the same strain, it is still necessary to conduct the fusibility experiments (Figure 5). However, the fusibility experiments require gemmules to be available and are very time-consuming. Our method provides a quicker way to determine which specimens have the potential to be genetically similar enough to be used further as one sample.

Even many structural genes are present in various copies in a genome of a sponge [25]. This variance is further increased by different allelic variants. Since the amount of material available from one individual tends to be small, as sponges grow slowly, it would be necessary to combine material from different individuals to gain material for analysing an enzyme of interest. Using our method can help reduce the disorienting heterogeneity that arises from combining random samples even from the same sampling site since individuals similar enough on the genetic level (close relatives) harbour a limited amount of allelic variants of the structural genes. The heterogeneity revealed in this work further emphasizes the need to determine the homogeneity of samples being used.

The proportion model method was developed for the analysis of ITS sequences and proved to be useful for distinguishing individuals of *E. fluviatilis*; however the underlying principle can be used for low copy number genes, as well. If a sample yields a sequence electropherogram displaying a double peak, the relative content of sequence variants having one or another nucleotide in that position can be assessed by sequencing a few clones to ascertain the respective intrinsic intensity values for both nucleotides, and comparing those to the values from the initial electropherogram. The accuracy of the results (around ±10%) would allow anyone to decide if the sequence variants are present either in 1∶3 or 2∶2 ratio, for example. For indels the same modelling approach can be used with higher precision.

## Supporting Information

File S1 **Direct sequencing electropherograms.** Catalogue names in the archive refer to the specimens specified in Materials and Methods. PCR amplifications were performed with 100 ng of DNA if not indicated otherwise in the trace name. Experimental details for specimens V2 and V3 are explained in text.
(ZIP)
